# ﻿Morphological and phylogenetic analyses reveal two new *Alternaria* species (Pleosporales, Pleosporaceae) in *Alternaria* section from Cucurbitaceae plants in China

**DOI:** 10.3897/mycokeys.107.124814

**Published:** 2024-07-22

**Authors:** Sein Lai Lai Aung, Feng-Yin Liu, Ya-Nan Gou, Zin Mar Nwe, Zhi-He Yu, Jian-Xin Deng

**Affiliations:** 1 Department of Plant Protection, College of Agriculture, Yangtze University, Jingzhou 434025, China Yangtze University Jingzhou China; 2 MARA Key Laboratory of Sustainable Crop Production in the Middle Reaches of the Yangtze River (Co-Construction by Ministry and Province), Yangtze University, Jingzhou 434025, China Yangtze University Jingzhou China; 3 Department of Applied Microbiology, College of Life Sciences, Yangtze University, Jingzhou 434025, China Yangtze University Jingzhou China

**Keywords:** Morphology, novel species, phylogeny, small-spored *Alternaria*, taxonomy

## Abstract

*Alternaria* species are commonly found as saprophytes, endophytes and plant pathogens. During a survey of small-spored *Alternaria* in China, two new species were discovered from Cucurbitaceae plants collected in Hubei and Sichuan provinces. This study identified two new species of *Alternaria* using seven genes (ITS, *GAPDH*, *TEF1*, *RPB2*, *Alt a 1*, *EndoPG*, and OPA10-2) for phylogenetic analyses and morphological characteristics. The two new species *A.jingzhouensis* and *A.momordicae* were described and illustrated. *Alternariajingzhouensis***sp. nov.**, associated with *Citrulluslanatus*, is characterized by producing muriform, ellipsoidal, flask-shaped, rostrate, and beaked conidia. It differs from *A.koreana*, *A.ovoidea*, and *A.baoshanensis* by bearing conidia in a simple conidiogenous locus with occasionally longer beaks in a chain, and from *A.momordicae***sp. nov.** by having shorter beaks. *Alternariamomordicae***sp. nov.** from *Momordicacharantia* was distinct from *A.koreana*, *A.ovoidea*, and *A.baoshanensis* by producing muriform, long ellipsoid or ovoid to obclavate, sometimes inverted club-shaped conidia on a single conidiogenous locus with a wider body and longer beak in a chain, and distinct from *A.jingzhouensis***sp. nov.** by a longer beak conidia. These two species were clearly distinguished from other species in the section Alternaria based on DNA based phylogeny and morphological characteristics. The morphological features were discussed and compared to relevant species in the present paper.

## ﻿Introduction

The Cucurbitaceae, also called cucurbits or the gourd family, consists of approximately 975 species belonging to 98 genera (Xu and Chang 2017). There are 35 genera with 151 species in China (Raven and Wu 2022). This family includes highly nutritious vegetables with significant economic value, such as cucumber, pumpkin, and so on. Watermelon (*Citrulluslanatus* (Thunb.) Matsum. & Nakai) is a popular fruit worldwide, and its seeds contain high levels of proteins, lipids and medicinal properties (Wani et al. 2011, Maoto et al. 2019). China is the world’s leading producer of watermelons (Qiang et al. 2024). Bitter gourd (*Momordicacharantia* L.) is normally cultivated in China for its fruit as a popular vegetable and traditional medicine (Sun et al. 2023). *Alternaria*-like leaf blight can severely affect the crop production of Cucurbitaceae (Maheswari and Sankaralingam 2010; Ma et al. 2021). Many *Alternaria* species have been reported to be associated with cucurbit plants, including *A.alternata* (Fr.) Keissl. (Chen et al. 1993; Zhao et al. 2016a, 2016b; Ma et al. 2021), *A.baoshanensis* J.F. Li, Phookamsak & Jeewon (Li et al. 2023), *A.brassicae* (Berk.) Sacc. (Simmons 2007), A.brassicaevar.nigrescens (Peglion) Sacc. & Traverso (Simmons 2007), *A.caudata* Cooke & Ellis (Simmons 2007), *A.cucumericola* E.G. Simmons & C.F. Hill (Simmons 2007), *A.cucumerina* (Ellis & Everh.) J.A. Elliott (Chen et al. 1993; Zhang 2003; Simmons 2007; Ma et al. 2021), *A.cylindrorostra* T.Y. Zhang (Zhang 2003; Simmons 2007), *A.gaisen* Nagano ex Bokura (Ma et al. 2021), *A.granulosa* (Bubák) E.G. Simmons (Simmons 2007), *A.hydrangeae* D. F. Pei & J. X. Deng (Liu et al. 2022), *A.infecotria* E.G. Simmons (Ma et al. 2021), *A.loofahae* E.G. Simmons & Aragaki (Simmons 2007), *A.nigrescens* (Peglion) Neerg. (Simmons 2007), *A.peponicola* (Rabenh.) E.G. Simmons (Zhang 2003; Simmons 2007), *A.peponis* Yatel (Simmons 2007), and *A.tenuissima* (Kunze) Wiltshire (Chen et al. 1993; Zhao et al. 2016a, 2016b; Ma et al. 2021).

The genus *Alternaria*Nees von Esenbeck (1816) is categorized according to its morphological characteristics, typified by *A.alternata* with muriform and catenulate conidia (Simmons 2007). Simmons (1992) applied standard criteria to achieve solid taxonomic outcomes for *Alternaria* species, primarily relying on the sporulation patterns and developmental morphology of conidia. In 2007, Simmons illustrated approximately 276 species (148 large-spored species and 128 small-spored species) and provided a final summary of morphological taxonomy on *Alternaria*. The small-spored species fall into 10 subsections containing the type species of *A.alternata* (Simmons 2007). In 2003, Zhang identified approximately 80 small-spored species associated with specific host plant families in China.

To date, the utilization of multigene phylogenetic analyses has played a crucial role in understanding the *Alternaria* genus (Pryor and Gilbertson 2000; Pryor and Bigelow 2003; Hong et al. 2005; Runa et al. 2009; Woudenberg et al. 2013, 2014; Lawrence et al. 2013, 2014, 2016; Poursafar et al. 2018). The genus contains 24 internal clades (sections) and six monotypic lineages (Woudenberg et al. 2013) using type or referenced strains collected by Simmons (2007), which has recently been updated to 29 sections (Li et al. 2023). Small-spored *Alternaria* species are also frequently isolated from Cucurbitaceae in China (Ma et al. 2021). Woudenberg et al. (2015) provided a clear and stable species classification of section Alternaria based on the genomic and multi-loci analyses, from which the species commonly produce concatenated conidia (Norphanphoun et al. 2021; Li et al. 2022; Gou et al. 2022). Consequently, the combination of morphology and molecular techniques provides a better understanding of species in section Alternaria (Aung et al. 2020).

During the investigation of small-spored *Alternaria* species in China, two new taxa were isolated from gourd plants of *Citrulluslanatus* and *Momordicacharantia*. The aim of this study was to characterize and differentiate both taxa using morphology and multigene sequence analyses. This research sought to enhance understanding of *Alternaria* species diversity within the Cucurbitaceae family, offering crucial taxonomic information for species conservation efforts.

## ﻿Materials and methods

### ﻿Isolation

Leaves of *Citrulluslanatus* and *Momordicacharantia* with necrotic spots were collected from Jingzhou, Hubei in 2022 and Deyang City, Sichuan Province in 2016 China, respectively. To facilitate isolation, the specimens were carefully enclosed in sterile plastic bags and transported to the laboratory. Subsequently, the tissues were accurately divided into small segments, arranged on moist filter papers within Petri dishes, and incubated at 25 °C to promote spore production. After sporulation, spores of *Alternaria* were individually collected using sterilized glass needles under a stereo microscope (Shunyu SZM series) and transferred onto potato dextrose agar (PDA) plates. Each distinct culture was purified and preserved in test-tube slants maintained at 4 °C. Additionally, dried cultures derived from individual spores and reference strains were stored in the Fungi Herbarium of Yangtze University (YZU), located in Jingzhou, Hubei, China.

### ﻿Morphology

To study the features of colonies, the strains were grown on PDA at 25 °C for 7 days without light. To examine the characteristics of the conidia (size, shape, sporulation, etc.), fresh mycelia were transferred to potato carrot agar (PCA) and V8 juice agar (V8A) plates and then placed in an incubator at 22 °C with an 8-hour light cycle for 7 days (Simmons 2007). A total of 50 conidia were randomly selected and photographed for the morphological determination after mounting the conidia into lactophenol picric acid under an ECLIPSE Ni-U microscope system (Nikon, Japan). The sporulation patterns and morphological characteristics were also recorded.

### ﻿DNA extraction, PCR amplification and sequencing

Fresh mycelia growing on PDA were used to extract genomic DNA with the CTAB method, as described by Watanabe et al. (2010). To amplify multigene fragments, including the internal transcribed spacer rDNA region (ITS), glyceraldehyde-3-phosphate dehydrogenase (*GAPDH*), translation elongation factor 1 alpha (*TEF1*), RNA polymerase second largest subunit (*RPB2*), *Alternaria* major allergen gene (*Alt a 1*), endopolygalacturonase gene (*EndoPG*), and an anonymous gene region (OPA10-2), primer pairs were employed including ITS5/ITS4 (White et al. 1990), gpd1/gpd2 (Berbee et al. 1999), EF1-728F/EF1-986R (Carbone and Kohn 1999), RPB2-5F/RPB2-7cR (Liu et al. 1999), Alt-for/Alt-rev (Hong et al. 2005), PG3/PG2b (Andrew et al. 2009) and OPA10-2L/OPA10-2R (Andrew et al. 2009), respectively. The PCR reaction mixture was 25 μL, including 21 μL of 1.1×Taq PCR Star Mix from TSINGKE, 2 μL of template DNA, and 1 μL of each primer. The amplification process was carried out in an Eppendorf Mastercycler, following the protocols outlined by Woudenberg et al. (2015). After a successful amplification, the PCR products were purified and sequenced by TSINGKE company (Beijing, China). The obtained sequences were assembled using BioEdit v. 7.2.3 (Hall 1999) and primarily aligned with PHYDIT v.3.2 (Chun 1995) then deposited into GenBank (https://www.ncbi.nlm.nih.gov/) (Table 1).

**Table 1.** *Alternaria* strains used in this study and their GenBank accession numbers.

Species	Strain	Host/Substrate	Country	GenBank accession numbers						
				ITS	*GAPDH*	*TEF1*	*RPB2*	*Alt a 1*	*EndoPG*	OPA10-2
*A.alternantherae*	CBS 124392	*Solanummelongena*	China	KC584179	KC584096	KC584633	KC584374	KP123846	np	np
*A.alternata*	CBS 916.96T	*Arachishypogaea*	India	AF347031	AY278808	KC584634	KC584375	AY563301	JQ811978	KP124632
	CBS 106.34T	*Linumusitatissimum*	Unknown	Y17071	JQ646308	KP125078	KP124771	KP123853	KP124000	KP124608
	CBS 102596T	*Citrusjambhiri*	USA	KP124328	KP124183	KP125104	KP124796	KP123877	KP124030	KP124637
	CBS 121336T	*Allium* sp.	USA	KJ862254	KJ862255	KP125141	KP124833	KJ862259	KP124067	KP124676
	CBS 121547T	*Pyrusbretschneideri*	China	KP124372	KP124224	KP125150	KP124842	KP123920	KP124076	KP124685
	CBS 119543T	*Citrusparadisi*	USA	KP124363	KP124215	KP125139	KP124831	KP123911	KP124065	KP124674
	CBS 918.96R	*Dianthuschinensis*	UK	AF347032	AY278809	KC584693	KC584435	AY563302	KP124026	KP124633
	CBS 127671T	*Stanleyapinnata*	USA	KP124381	KP124233	KP125159	KP124851	KP123929	KP124085	KP124694
	CBS 121455T	*Broussonetiapapyrifera*	China	KP124368	KP124220	KP125146	KP124838	KP123916	KP124072	KP124681
	CBS 117.44T	*Godetia* sp.	Denmark	KP124303	KP124160	KP125079	KP124772	KP123854	KP124001	KP124609
	CBS 127672T	*Astragalusbisulcatus*	USA	KP124382	KP124234	KP125160	KP124852	KP123930	KP124086	KP124695
	CBS 102.47R	*Citrussinensis*	USA	KP124304	KP124161	KP125080	KP124773	KP123855	KP124002	KP124610
	CBS 102599T	*Minneolatangelo*	Turkey	KP124330	KP124185	KP125106	KP124798	KP123879	KP124032	KP124639
	CBS 102595T	*Citrusjambhiri*	USA	FJ266476	AY562411	KC584666	KC584408	AY563306	KP124029	KP124636
	CBS 103.33T	Soil	Egypt	KP124302	KP124159	KP125077	KP124770	KP123852	KP123999	KP124607
*A.arborescens*	CBS 126.60	Wook	UK	KP124397	KP124249	KP125175	KP124867	JQ646390	KP124101	KP124710
	CBS 119545T	*Senecioskirrhodon*	New Zealand	KP124409	KP124260	KP125187	KP124879	KP123956	KP124113	KP124723
	CBS 101.13T	Peat soil	Switzerland	KP124392	KP124244	KP125170	KP124862	KP123940	KP124096	KP124705
	CBS 105.24	*Solanumtuberosum*	Unknown	KP124393	KP124245	KP125171	KP124863	KP123941	KP124097	KP124706
	CBS 119544T	*Avenasativa*	New Zealand	KP124408	JQ646321	KP125186	KP124878	KP123955	KP124112	KP124722
	CBS 105.49	Contaminant blood culture	Italy	KP124396	KP124248	KP125174	KP124866	KP123944	KP124100	KP124709
	CBS 112749	*Malusdomestica*	South Africa	KP124401	KP124253	KP125179	KP124871	KP123948	KP124105	KP124715
*A.baoshanensis*	MFLU 21-0124T	*Curcubitamoschata*	China	MZ622003	OK236706	OK236613	OK236659	OK236760	np	np
	MFLU 21-0296	*C.moschata*	China	MZ622004	OK236707	OK236612	OK236660	OK236759	np	np
*A.breviconidiophora*	MFLUCC 21-0786T	*Digitalis* sp.	Italy	MZ621997	OK236698	OK236604	OK236651	OK236751	np	np
*A.burnsii*	CBS 118817T	*Tinosporacordifolia*	India	KP124424	KP124274	KP125202	KP124893	KP123971	KP124128	KP124738
	CBS 118816T	*Rhizophoramucronata*	India	KP124423	KP124273	KP125201	KP124892	KP123970	KP124127	KP124737
*A.ellipsoidialis*	MFLUCC 21-0132T	*Brassica* sp.	Italy	MZ621989	OK236690	OK236596	OK236643	OK236743	np	np
*A.eupatoriicola*	MFLUCC 21-0122T	*Eupatoriumcannabinum*	Italy	MZ621982	OK236683	OK236589	OK236636	OK236736	np	np
*A.falcata*	MFLUCC 21-0123T	*Atriplex* sp.	Italy	MZ621992	OK236693	OK236599	OK236649	OK236746	np	np
*A.gaisen*	CBS 632.93R	*Pyruspyrifolia*	Japan	KC584197	KC584116	KC584658	KC584399	KP123974	AY295033	KP124742
	CBS 118488R	*P.pyrifolia*	Japan	KP124427	KP124278	KP125206	KP124897	KP123975	KP124132	KP124743
*A.gossypina*	CBS 102601T	*Minneolatangelo*	Colombia	KP124433	KP124282	KP125212	KP124903	KP123979	KP124138	KP124749
	CBS 104.32T	*Gossypium* sp.	Zimbabwe	KP124430	JQ646312	KP125209	KP124900	JQ646395	KP124135	KP124746
*A.jacinthicola*	CBS 878.95	*Arachishypogaea*	Mauritius	KP124437	KP124286	KP125216	KP124907	KP123983	KP124142	KP124753
	CBS 133751T	*Eichhorniacrassipes*	Mali	KP124438	KP124287	KP125217	KP124908	KP123984	KP124143	KP124754
***A.jingzhouensis* sp. nov.**	**YZU 221144T**	***Citrulluslanatus***	**China**	**OR883772**	**OR887690**	**OR887686**	**OR887688**	**OR887694**	**OR887692**	**OR887684**
	**YZU 221145**	***C.lanatus***	**China**	**OR901948**	**OR914170**	**OR914166**	**OR914168**	**OR914174**	**OR914172**	**OR914176**
*A.koreana*	SPL2-1T	*Atractylodesovata*	Korea	LC621613	LC621647	LC621715	LC621681	LC631831	LC631844	LC631857
	SPL2-4	*A.ovata*	Korea	LC621615	LC621649	LC621717	LC621683	LC631832	LC631845	LC631858
*A.longipes*	CBS 121333R	*Nicotianatabacum*	USA	KP124444	KP124293	KP125223	KP124914	KP123990	KP124150	KP124761
	CBS 540.94R	*N.tabacum*	USA	AY278835	AY278811	KC584667	KC584409	AY563304	KP124147	KP124758
*A.minimispora*	MFLUCC 21-0127T	*Citrulluslanatus*	Thailand	MZ621980	OK236705	OK236587	OK236634	OK236734	np	np
***A.momordicae* sp. nov.**	**YZU 161378T**	***Momordicacharantia***	**China**	**OR883774**	**OR887691**	**OR887687**	**OR887689**	**OR887695**	**OR887693**	**OR887685**
	**YZU 161379**	***M.charantia***	**China**	**OR901949**	**OR914171**	**OR914167**	**OR914169**	**OR914175**	**OR914173**	**OR914177**
*A.muriformispora*	MFLUCC 21-0784T	*Plantago* sp.	Italy	MZ621976	OK236677	OK236583	OK236630	OK236730	np	np
*A.obpyriconidia*	MFLUCC 21-0121T	*Viciafaba*	Italy	MZ621978	OK236680	OK236585	OK236633	OK236732	np	np
*A.ovoidea*	MFLUCC 0782T	*Dactylisglomerata*	Italy	MZ622005	OK236708	OK236614	OK236661	OK236761	np	np
	MFLU 21- 0298	*D.glomerata*	Italy	MZ622006	OK236709	OK236615	OK236662	OK236762	np	np
*A.orobanches*	MFLUCC 21-0137T	*Orobanche* sp.	Italy	MZ622007	OK236710	np	np	OK236763	np	np
	MFLU 21-0303	*Orobanche* sp.	Italy	MZ622008	OK236711	np	np	OK236764	np	np
*A.phragmiticola*	MFLUCC 21-0125T	*Phragmites* sp.	Italy	MZ621994	OK236696	OK236602	OK236649	OK236749	np	np
*A.rostroconidia*	MFLUCC 21-0136T	*Arabis* sp.	Italy	MZ621969	OK236670	OK236576	OK236623	OK236723	np	np
*A.salicicola*	MFLUCC 22-0072T	*Salixalba*	Russia	MZ621999	OK236700	OK236606	OK236653	OK236753	np	np
*A.tomato*	CBS 103.30	*Solanumlycopersicum*	Unknown	KP124445	KP124294	KP125224	KP124915	KP123991	KP124151	KP124762
	CBS 114.35	*S.lycopersicum*	Unknown	KP124446	KP124295	KP125225	KP124916	KP123992	KP124152	KP124763
*A.torilis*	MFLUCC 14-0433T	*Torilisarvensis*	Italy	MZ621988	OK236688	OK236594	OK236641	OK236741	np	np

Notes: Novel species proposed in this study are marked in bold. Ex-type strains are marked ‘T’. Representative strains are marked ‘R’. No products are ‘np’.

### ﻿Phylogenetic analyses

Preliminary BLAST searches on the National Center for Biotechnology Information (NCBI) website (https://blast.ncbi.nlm.nih.gov/Blast.cgi) indicated that the current species are highly similar to species within the *Alternaria* genus. Subsequently, sequence data of 57 *Alternaria* strains and *A.alternantherae* Holcomb & Antonop. CBS 124392 (outgroup) were retrieved from the GenBank database and referenced from relevant publications (Woudenberg et al. 2015; Li et al. 2022; Romain et al. 2022) (Table 1). The gene sequences were concatenated and edited manually with equal weight in MEGA v.11.0.13 (Tamura et al. 2021), and gaps were treated as missing data. Bayesian inference (BI) analysis was carried out using MrBayes v. 3.2.6 (Ronquist et al. 2012). This analysis employed a Markov Chain Monte Carlo (MCMC) algorithm to estimate Bayesian posterior probabilities. The best-fit evolutionary model (GTR+I+G) was determined using MrModeltest v. 2.3 (Nylander 2004, Posada and Crandall 1998) with the Akaike Information Criterion (AIC). In MrModeltest, the file "MrModelblock″ was executed in the PAUP path (Swofford 2002) and the MrMt path (Nylander 2004). Bayesian analyses included two parallel runs for 10,000,000 generations (ngen) with the stop rule option and a sampling frequency set to every 100 generations (samplefreq=100). The run was stopped when the standard deviation of split frequencies reached a value below 0.01. The first 25% of sampled trees were discarded as burn-in. Additionally, a maximum likelihood (ML) analysis was performed using RAxML v.7.0.3 (Stamatakis et al. 2008). The GTRGAMMAI model was implemented using ML+ rapid bootstrap setting with 1000 replications to assess branch support. The tree was visualized with FigTree v1.4.3 (Rambaut 2016). Nodes in the phylogram displayed branch support values equal to or above 0.60/60% for posterior probability (PP)/bootstrap (BS) values.

## ﻿Results

### ﻿Phylogenetic analyses

**Figure 1.**
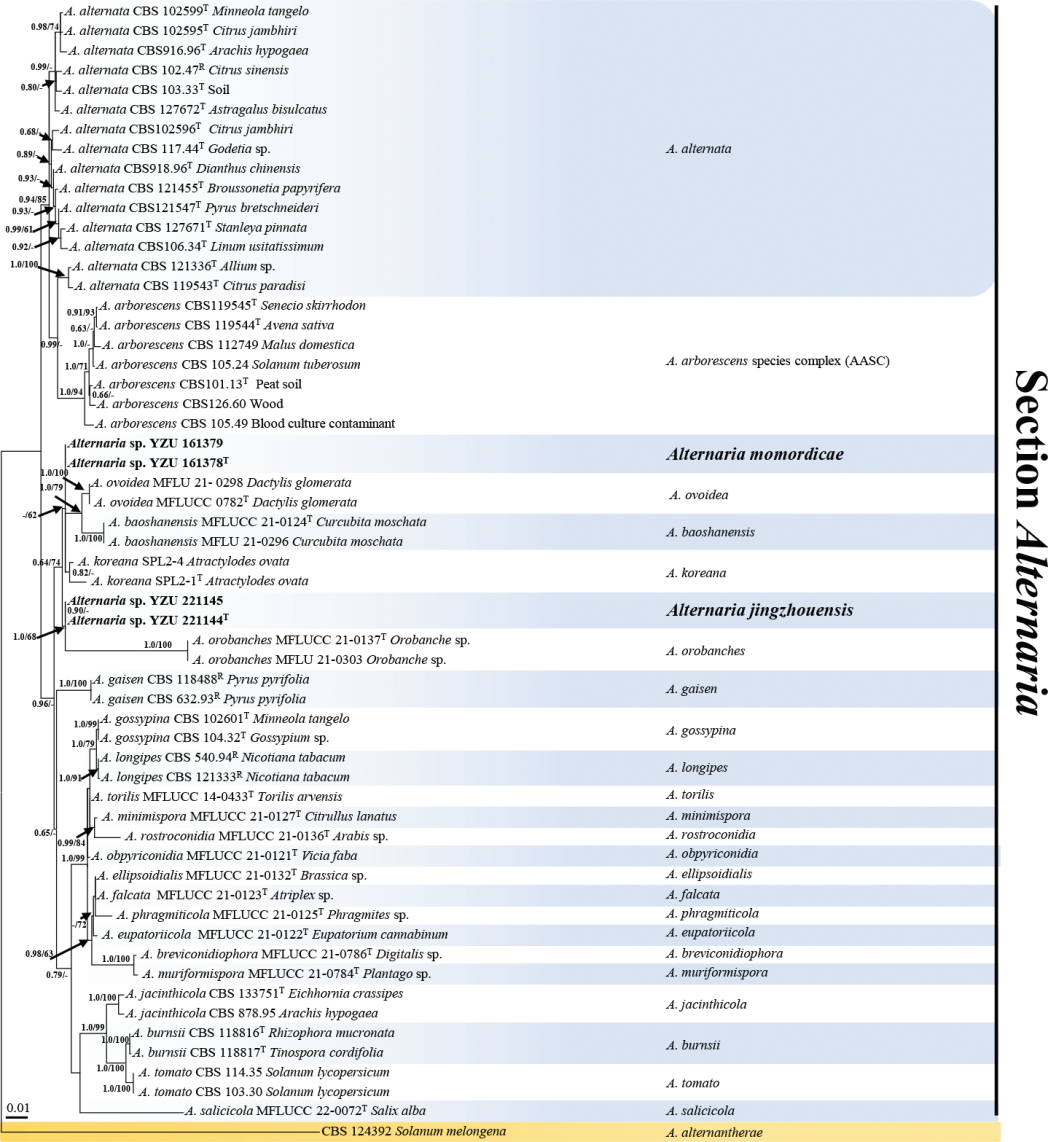
Phylogenetic tree of the *Alternaria* species most related to the new taxa based on maximum likelihood analysis using the combined gene sequences of ITS, *GAPDH*, *TEF1*, *RPB2*, *Alt a 1*, *EndoPG* and OPA10-2 which rooted with *Alternariaalternantherae* (CBS 124392) from sect. Alternantherae. The Bayesian posterior probabilities >0.60 (PP) and bootstrap support values >60 (BS) are given at the nodes (PP/BS). The novel species are highlighted in bold. Ex-type isolates are marked with a superscript T and Representative isolates are marked with a superscript R.

The dataset includes a total of 58 *Alternaria* strains with 3627 characters in total after alignment. The dataset consists of 533 characters for ITS, 574 for *GAPDH*, 216 for *TEF1*, 757 for *RPB2*, 444 for *EndoPG*, 469 for *Alt a 1*, and 634 for OPA10-2. Both Bayesian inference (BI) and maximum likelihood (ML) analyses yielded similar topologies. The ML tree was selected for discussing the placement of our new species (Fig. 1). The results indicated that all *Alternaria* strains in the present study fell into Alternaria section with PP values of 1.0. The present four strains separated into two individual clades sister to *A.koreana* O. Hassan, B.B.N.D. Romain, J.S. Kim & T. Chang, *A.ovoidea* J.F. Li, Camporesi, Bhat & Phookamsak, *A.baoshanensis*, and *A.orobanches* J.F. Li, Camporesi, Phookamsak & Jeewon (Bayesian posterior probability (BI-BPP)/Maximum-Likelihood bootstrap proportions (ML-BS) = 0.64/74%).

The clade containing YZU 161378 and YZU 161379 was closely related to *A.baoshanensis*, *A.koreana*, *A.ovoidea*, and forming a distinct branch. While another clade, YZU 221144 and YZU 221145 was found to be independent with a posterior probability (PP) of 1.00 and bootstrap (BS) values of 68%, and it was closely related to *A.orobanches*. These results suggest that the present strains represent two new taxa.

### ﻿Taxonomy

#### ﻿ Alternaria jingzhouensis

Taxon classificationFungiPleosporalesPleosporaceae

S.L.L. Aung & J.X. Deng sp. nov.

D4E3DDD8-26E9-5A6C-969E-6CC03CAF933F

MycoBank No: 851272

Fig. 2

##### Type.

China, Hubei Province, Jingzhou city, Yangtze University (west campus) on infected leaves of *Citrulluslanatus* 2022, F.Y Liu, (YZU-H-2022030, holotype), ex-type culture YZU 221144.

##### Etymology.

Named after the collecting locality, Jingzhou (Hubei, China)

##### Description.

**Figure 2.**
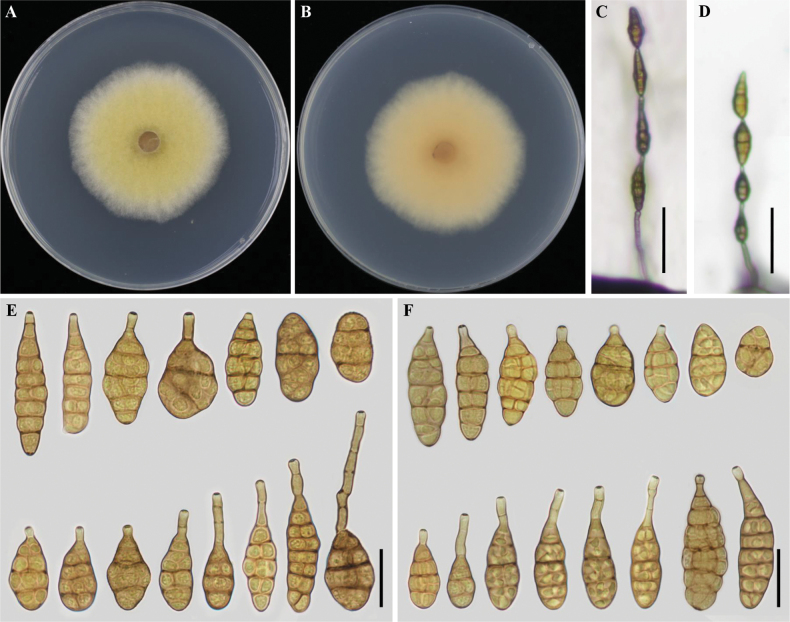
*Alternariajingzhouensis* sp. nov. (ex-type YZU 221144) **A, B** seven-day-old culture on PDA**C, D** conidiophores and conidia on PCA and V8A, respectively **E, F** conidia on PCA and V8A, respectively. Scale bars: 25 μm (**E, F**); 50 μm (**C, D**).

***Colonies*** on PDA (7 d at 25 °C) pale luteous to amber in the center, white at the edges, light to moderate rosy buff or pale saffron in reverse, cottony surface and 49–52 mm in diam., at 25 °C for 7 days (Fig. 2A, B). On PCA (7 d at 22 °C), ***conidiophores*** arising from substrate, simple, straight or flexuous, light to olivaceous buff, 41–99 (–151) × 3.5–5 μm (x̄ = 73 × 4.4 µm, n = 20), ***conidiogenous cells*** 5–11 × 3–6 µm (x̄ = 8 × 4 µm, n = 20), mono- to polytretic, terminal, determinate, cylindrical, olivaceous buff, smooth, thin-walled, apically doliiform, with 1 conidiogenous locus cicatrized on conidial secession, sometimes swollen near conidiogenous loci; ***conidia*** 3–5 units per chain, arising from the apex or near the apex of the conidiophores or terminal hyphae, muriform, ellipsoidal, flask-shaped, rostrate, beaked, 28–51 × 11–21 μm (x̄ = 38 × 16.4, n = 50), with 1–4 transverse septa with 0–2 branching (Fig. 2C, E); On V8A (7 d at 22 °C), ***conidiophores*** 40–94 × 4–7 μm (x̄ = 58 × 5, n = 20), simple, straight or flexuous, light to olivaceous buff; ***conidiogenous cells*** 5–13 × 3–6 µm (x̄ = 8 × 4 µm, n = 20), mono- to polytretic, terminal, determinate, cylindrical, olivaceous buff, smooth, thin-walled, apically doliiform, with 1 conidiogenous locus, sometimes swollen near conidiogenous loci cicatrized on conidial secession; ***conidia*** 3–5 units per chain, arising from the apex or near the apex of the conidiophores or terminal hyphae, muriform, ellipsoidal, flask-shaped, rostrate, beaked, 22–51 × 3–16 μm (x̄ = 33.9×13.2, n = 50), 1–6 transverse septa with 0–2 branching (Fig. 2D, F).

##### Additional isolate examined.

China, Hubei Province, Jingzhou city, Yangtze University (west campus) on infected leaves of *Citrulluslanatus* 2022, F.Y Liu, living culture YZU 221145.

##### Notes.

Phylogenetically, *A.jingzhouensis* sp. nov. is different from its sister species *A.baoshanensis*, *A.koreana*, *A.momordicae* sp. nov., *A.orobanches* and *A.ovoidea* based on sequences derived from seven genes (Fig. 1). After conducting a nucleotide pairwise comparison as recommended by Jeewon and Hyde (2016), the present species can be readily distinguished from the closet species *A.koreana*, *A.momordicae* sp. nov. and *A.orobanches* constructed on any of the ITS, *GAPDH*, *TEF1*, *RPB2*, *Alt a 1*, *EndoPG*, and OPA10-2 genes, which has 1 bp difference in the ITS region, 1 bp in *GAPDH*, 1 bp in *TEF1*, 7 pb in *RPB2*, 9 bp in *Alt a1*, 10 bp in *EndoPG*, and 4 bp in OPA10-2 when compared with *A.koreana*, 1 bp in *GAPDH*, 4 bp in *RPB2*, and 11 bp in OPA10-2 when compared with *A.momordicae* sp. nov. and 49 bp differences in the ITS region when compared with sister species *A.orobanches*. Morphologically, the species is distinct from *A.baoshanensis*, *A.koreana*, and *A.ovoidea* as it produces conidia on a simple conidiogenous locus with occasionally longer beaks in a chain of 3–5 units, and from *A.momordicae* sp. nov. by having shorter beaks (Table 2).

**Table 2.** Conidial features of the novel *Alternaria* species proposed here and their closest relatives in section Alternaria.

Species	Conidia				Conidia per chain	Medium	Reference
	Shape	Body (µm)	Beak (µm)	Septa			
*A.baoshanensis*	Subglobose to ellipsoidal, or subcylindrical to obpyriform	25–60 × 12–22	Short beak	3–6	1–3	PCA	Li et al. (2023)
***A.jingzhouensis* sp. nov.**	**Ellipsoidal, flask-shaped, rostrate, beaked**	**28–51 × 11–21**	**2–7(–15)**	**1–4**	**3–5**	**PCA**	**Present study**
		**22–51 × 3–16**	**3–7**	**1–6**	**3–5**	**V8A**	**Present study**
*A.koreana*	Obovate to long ellipsoid	12.9–61.2×8.6–20.7	4.5–9.1	2–8	1–2	SNA	Romain et al. (2022)
***A.momordicae* sp. nov.**	**Obclavate, inverted club-shaped**	**6–42 × 4–34**	**2–19.5**	**1–5**	**3–4**	**PCA**	**Present study**
		**24–61 × 10–17**	**3–25.**5	**1–5**	**3–4**	**V8A**	**Present study**
*A.orobanches*	Obclavate to ovoid	20–50 × 10–20	–	3–6	1–2	PCA	Li et al. (2023)
*A.ovoidea*	Ovoid	48–65 × 15.5–30	–	1–3	1	PDA	Li et al. (2022)

#### ﻿ Alternaria momordicae

Taxon classificationFungiPleosporalesPleosporaceae

S.L.L. Aung & J.X. Deng sp. nov.

40F7E142-199A-5257-84B7-0C9E5518C20F

MycoBank No: 851270

Fig. 3

##### Type.

China, Sichuan Province, Deyang city infected leaves of *Momordicacharantia*. 2016, J.X Deng, (YZU-H-2016001, holotype), ex-type culture YZU 161378.

##### Etymology.

Refers to the host genus, *Momordica*.

##### Description.

**Figure 3.**
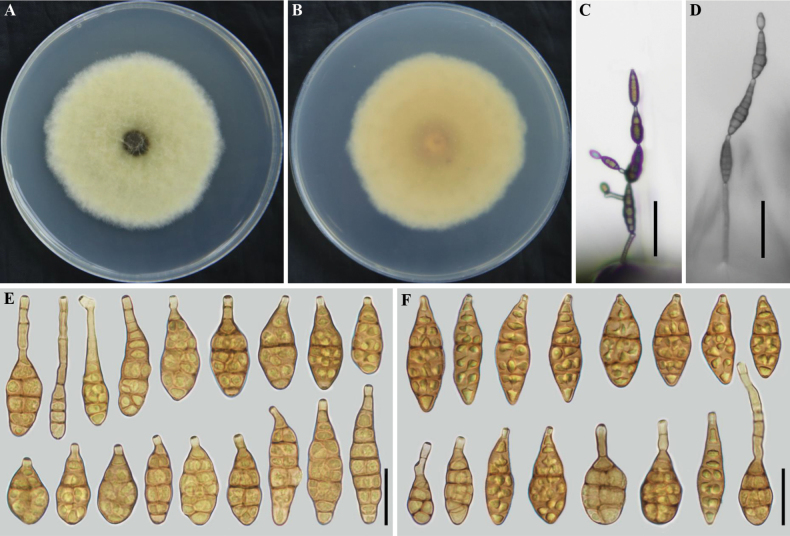
*Alternariamomordicae* sp. nov. (ex-type YZU 161378) **A, B** seven-day-old culture on PDA**C, D** conidiophores and conidia on PCA and V8A, respectively **E, F** conidia on PCA and V8A, respectively. Scale bars: 25 μm (**E, F**); 50 μm (**C, D**).

***Colonies*** on PDA (7 d at 25 °C) greyish yellow-green, light white at the edge, buff to salmon in reverse, surface compact, 50–55 mm in diam. (Fig. 3A, B). On PCA (7 d at 22 °C), ***conidiophores*** arising from substrate, simple, straight or flexuous, septate, olivaceous buff to olivaceous, 26.5–93 × 3–4 μm (x̄ = 59.5× 3.8 μm, n = 20); ***conidiogenous cells*** 5–10 × 3–5 µm (x̄ = 7 × 4 µm, n = 20), mono- to polytretic, terminal, determinate, cylindrical, olivaceous buff to olivaceous, smooth, thin-walled, apically doliiform, with 1 conidiogenous locus cicatrized on conidial secession, sometimes swollen near conidiogenous loci; ***conidia*** 3–4 units per chain, arising from the apex or near the apex of the conidiophores or terminal hyphae, muriform, long ellipsoid or ovoid to obclavate, sometime inverted club-shaped, 6–42 × 4–34 μm (x̄ = 32.8 × 13.5 μm, n = 50), 1–5 transverse septa, apical beak 2–19.5 μm long and 1–2 septa (Fig. 3C, E); On V8A(7 d at 22 °C), ***conidiophores*** straight or curved, smooth-walled, olivaceous buff 23–63(–208) × 3–5 μm (x̄ = 64.9 × 4.2 μm, n = 20); ***conidiogenous cells*** 5–13 × 3–4 µm (x̄ = 7 × 4 µm, n = 20), mono- to polytretic, terminal, determinate, cylindrical, olivaceous buff, smooth, thin-walled, apically doliiform, with 1 conidiogenous locus cicatrized on conidial secession, sometimes swollen near conidiogenous loci; ***conidia*** 3–4 units per chain, muriform, long ellipsoid or ovoid to obclavate, inverted club-shaped, 24–61×10–17 μm (x̄ = 39 × 14.3 μm, n = 50), 1–5 transverse septa with apical beak 3–25.5 μm long and 1–2 septa (Fig. 3D, F).

##### Additional isolate examined.

China, Sichuan Province, Deyang city infected leaves of *Momordicacharantia*. 2016, J.X Deng, living culture YZU 161379.

##### Notes.

After the combined dataset of ITS, *GAPDH*, *TEF1*, *RPB2*, *Alt a 1*, *EndoPG* and OPA10-2 gene fragments, *A.momordicae* sp. nov. is readily distinguished from its sister species *A.baoshanensis*, *A.jingzhouensis* sp. nov., *A.koreana*, and *A.ovoidea*, (Fig. 1). After a nucleotide pairwise comparison as suggested by Jeewon and Hyde (2016), the present species can be readily distinguished from the closet species *A.koreana* and others related a novel species based on any of the ITS, *GAPDH*, *TEF1*, *RPB2*, *Alt a 1*, *EndoPG*, and OPA10-2 genes, which has 1 bp difference in the ITS region, 1 bp in *GAPDH*, 1 bp in *TEF1*, 4 bp in *RPB2*, 8 bp in *Alt a1* and 10 bp in *EndoPG* when compared with *A.koreana* and 1 bp in *GAPDH*, 4 bp in *RPB2*, and 11 bp in OPA10-2 when compared with *A.jingzhouensis* sp. nov.. Morphologically, *A.momordicae* sp. nov. produces conidia on PCA that are significantly shorter than those on V8A. It can be distinguished from *A.baoshanensis*, *A.koreana*, and *A.ovoidea* by producing conidia on a single conidiogenous locus with a wider body and longer beak in a chain of 3–4 units. Additionally, it differs from *A.jingzhouensis* sp. nov. by having a longer beak (Table 2).

## ﻿Discussion

Most of the *Alternaria* species published before the year 2000s relied on morphology to characterize the species status (Simmons 2007). In this study, two new *Alternaria* species, *A.jingzhouensis* and *A.momordicae*, have been identified and illustrated using the morphological method of Simmons (2007) and phylogenetic analysis of seven gene loci. Both resemble the type small-spored species of *A.alternata* in morphology but are easily distinguished by short chains, which also differentiate them from each other and their phylogenetically closely related species of *A.baoshanensis*, *A.koreana*, *A.ovoidea* and *A.orobanches* by the chain formation of sporulation patterns (Table 2). In recent publications, the *Alternaria* species descriptions have not followed the morphological standard created by Simmons (2007) (Romain et al. 2022; Li et al. 2022). Simmons (2007) classified the genus *Alternaria* into small-spored and large-spored taxa based on morphology. Andrew et al. (2009) noted that phylogenetic studies have confirmed a distinct separation between large- and small-spored *Alternaria* species. Woudenberg et al. (2015) identified 35 morphospecies as synonyms of *A.alternata*, but their relationships remain unclear due to inconsistencies and lack of detailed morphological information. Accurate identification and classification of species within these small-spored *Alternaria* species require strong identification through multigene sequence analysis (Kgatle et al. 2018). Li et al. (2023) described that recent studies using combined multi-locus phylogeny suggest that certain *A.alternata* species classified under section Alternaria may not constitute a monophyletic group in DNA sequence-based phylogenies. To reduce potential misidentification of morphological characteristics within this section, this study utilized PCA and V8A media for 7 days at 22 °C to identify *Alternaria* species, following Simmons’ (2007) recommendations. These media effectively promote typical morphological characteristics. Hence, it is strongly recommended to use the standard of morphological identification for further describing small-spored and large-spored *Alternaria* in order to reduce taxonomic ambiguity caused by different temperatures and substrates.

With the development of molecular studies, the species-group was re-defined and the section Alternaria was introduced and updated (Pryor and Gilbertson 2000; Lawrence et al. 2013; Woudenberg et al. 2013; Li et al. 2023). The section Alternaria is one of the small-spored *Alternaria* species groups and comprises 11 phylogenetic species and one species complex (Woudenberg et al. 2015). The two new Alternaria species are identified as members of section Alternaria according to the multigene sequence analysis of ITS, *GADPH*, *RPB2*, *TEF1*, *Alt a 1*, *EndoPG* and OPA10-2 gene sequences, which are close to *A.baoshanensis* (Li et al. 2023) from *Curcubitamoschata* (Cucurbitaceae), *A.koreana* (Romain et al. 2022) from *Atractylodesovata* (Compositae), *A.orobanches* (Li et al. 2023) from *Orobanche* sp. (Orobanchaceae), and *A.ovoidea* (Li et al. 2022) from *Dactylisglomerata* (Poaceae). Three genes, *GAPDH*, *RPB2*, and OPA10-2, provide more informative data for the classification of the current species.

Small-spored *Alternaria* species have been frequently reported on Cucurbitaceae plants worldwide, including *A.alternata* (Chen et al. 1993; Zhao et al. 2016a, 2016b; Ma et al. 2021), *A.baoshanensis* (Li et al. 2023), *A.caudata* (Simmons 2007), *A.gaisen* (Ma et al. 2021), *A.infecotria* (Ma et al. 2021), *A.peponicola* (Zhang 2003; Simmons 2007), and *A.tenuissima* (Chen et al. 1993; Zhao et al. 2016a, 2016b; Ma et al. 2021). The present two small-spored species, *A.jingzhouensis* sp. nov. and *A.momordicae* sp. nov., were first found on *C.lanatus* and *M.charantia*, respectively, in China. Pathogenicity tests were performed on detached and living leaves for the two new species, which showed weak pathogenicity (data not shown). However, they did exhibit a certain level of aggressiveness on cucurbit plants. The two species, *A.jingzhouensis* sp. nov. and *A.momordicae* sp. nov., were found to be non-pathogenic to their host plants, possibly due to their saprophytic or weakly pathogenic nature when encountering resistance from *C.lanatus* and *M.charantia*. These findings provide valuable insights into Alternaria leaf diseases in Cucurbitaceae.

## Supplementary Material

XML Treatment for Alternaria jingzhouensis

XML Treatment for Alternaria momordicae

## ﻿Additional information

### Author ORCIDs

Sein Lai Lai Aung https://orcid.org/0009-0006-2738-5598

Feng-Yin Liu https://orcid.org/0000-0003-3114-603X

Ya-Nan Gou https://orcid.org/0009-0005-1740-4065

Zin Mar Nwe https://orcid.org/0009-0000-6376-8306

Zhi-He Yu https://orcid.org/0000-0001-9477-4135

Jian-Xin Deng https://orcid.org/0000-0001-7304-5603

### Data availability

All of the data that support the findings of this study are available in the main text.
